# Novel association between asthma and osteoarthritis: a nationwide health and nutrition examination survey

**DOI:** 10.1186/s12890-021-01425-6

**Published:** 2021-02-16

**Authors:** Hyeon-Kyoung Koo, Pamela Song, Joo-Hyun Lee

**Affiliations:** 1 Division of Pulmonary and Critical Care Medicine, Department of Internal Medicine, Inje University Ilsan Paik Hospital, Goyang, Korea; 2 Department of Neurology, Inje University Ilsan Paik Hospital, Goyang, Korea; 3 Division of Rheumatology, Department of Internal Medicine, Inje University Ilsan Paik Hospital, Goyang, 10380 Korea

**Keywords:** Asthma, Osteoarthritis, COPD, KNHANES

## Abstract

**Background:**

Asthma and osteoarthritis (OA) are medical conditions that inhibit physical activity and adversely affect quality of life. Despite the high prevalence, there are limited studies focusing on the comorbid condition and association between asthma and OA. The aim of this study was to assess the prevalence of OA co-occurring with asthma and to identify the relevant clinical considerations.

**Methods:**

Adult participants aged over 40 years who completed questionnaire assessments and spirometry tests were enrolled from the Korean National Health and Nutrition Examination Survey. Asthma and OA were defined based on the medical history of a diagnosis made by a doctor. Radiographic severities of OA were measured using the Kellgren–Lawrence grading system. Chronic obstructive pulmonary disease (COPD), as a comparative respiratory disease, was diagnosed based on the spirometric results.

**Results:**

A total of 9344 subjects were enrolled, and the prevalence of asthma and COPD were 4.6% ± 0.3% and 12.0% ± 0.5%, respectively. The prevalence of OA in the asthma group was 31.9% ± 2.8%, which was significantly higher than that in the COPD (17.8% ± 1.5%) or control (16.2% ± 0.6%) groups. OA was more prevalent in patients with asthma after adjusting for age, sex, body mass index, and smoking status (OR 1.65; 95% CI 1.27–2.13). Furthermore, after adjustment of this model for the prescription of OA medication, OA remained independently associated with asthma (OR 1.56; 95% CI 1.10–2.20). Conversely, the relationship of OA medication with asthma was not significant (*P* = 0.64). This relationship was evident in patients with asthma without airflow limitation measured by spirometry (OR 1.97; 95% CI 1.32–2.93). Moreover, the radiographic severity of knee OA correlated with asthma (OR 1.10; 95% CI 1.0–‍‍1.21).

**Conclusions:**

OA shows a high prevalence in patients with asthma, higher than in patients with COPD or the controls. The comorbid characteristics of these two conditions need to be considered in clinical practice.

## Background

The World Health Organization estimates that approximately 300 million people globally have asthma [1–3]. The Global Initiative for Asthma reports that the prevalence of this disease ranges from 1 to 18% in different countries [3], and it accounts for about 1% of all disability-adjusted life years lost [4]. Asthma is characterized by chronic airway inflammation with variable airflow limitation, and factors such as genetics and environmental variations may contribute to its development [3]. Recent reports have suggested that patients with severe asthma who require systemic glucocorticoid therapy have a higher rate of comorbidities, including osteoporosis, functional dyspepsia, cataracts, and obstructive sleep apnea [5]. However, apart from atopic disease, the risk factors for and comorbidities related to asthma are largely unknown.

Osteoarthritis (OA) is a degenerative joint disease that causes structural joint damage and pain. In US adults aged 25 years or older, the prevalence of OA of the hand, hip, or knee joint increased from 21 million in 1995 to 27 million in just over a decade [6]. OA is prevalent in female patients, its prevalence increases with age (≥ 50 years), and it more frequently affects the joints of the hands and knees [6]. Older patients with joint pain have limited physical activity that indeed affects their quality of life (QOL) [7].

Owing to the high prevalence of asthma and OA, they are commonly encountered as comorbid conditions in clinical practice. However, studies aimed at identifying the association between asthma and OA are limited. The underlying etiologies of these two conditions differ, although they have synergistic effects in hindering physical activity. In OA and asthma, physical activity is limited due to joint pain and dyspnea, respectively; thus, both conditions lead to a deterioration in the QOL. However, this comorbidity may be under-recognized in those who prefer a sedentary lifestyle to cope with joint pain or dyspnea. Furthermore, there is concern that non-steroidal anti-inflammatory drugs (NSAIDs), which are commonly prescribed to patients with OA, may aggravate the symptoms of asthma [8]. Therefore, examining the prevalence and relationship of OA and asthma is essential to understand their clinical implications on aspects such as physical activity and QOL. This study aimed to determine the prevalence of OA co-occurring with asthma and to identify the characteristics of these comorbid conditions. The presence of OA was determined in healthy adults, patients with asthma, and patients with chronic obstructive pulmonary disease (COPD)—COPD was selected as a comparative respiratory disease of asthma. Data from the Korean National Health and Nutrition Examination Survey (KNHANES) were used for analyses.

## Methods

### Study design and participants

The KNHANES is a collection of nationally representative, cross-sectional, population-based health and nutritional surveys conducted by the Korean Centers for Disease Control and Prevention (KCDC) since 1998. The participants were selected using proportional allocation system sampling with multiple stratifications based on geographical area, age, and sex. The KNHANES includes a health interview, physical examination, laboratory tests, and nutritional questionnaire. All subjects who completed the questionnaire, laboratory tests, and a pulmonary function test were selected. The entire survey population participated voluntarily and provided written informed consent. The KNHANES protocol was approved by the institutional review board of the KCDC. The date is available under following web address (https://knhanes.cdc.go.kr/knhanes/sub03/sub03_02_05.do).

### Definition

The KNHANES includes a self-reported questionnaire including the following items for various diseases: “Have you been diagnosed with the disease by a doctor?” (Yes/No) and “Do you take medicine or treatment for the disease?” (Yes/No)—asthma and OA were defined based on the responses regarding the individual’s history of a medical diagnosis. Patients with asthma were divided into two groups according to the presence of airflow limitation on spirometry (forced expiratory volume in 1 s [FEV_1_: L]/forced vital capacity [FVC: L] < 0.7). COPD was defined when spirometry revealed airway obstruction (FEV_1_/FVC < 0.7) among adults ≥ 40 years of age without a history of asthma according to the Global Initiative for Chronic Obstructive Lung Disease (GOLD) guidelines [9]. Other comorbidities, including hypertension, diabetes, hypercholesterolemia, and obesity, were defined based on the KNHANES protocol. Hypertension was defined as a systolic blood pressure ≥ 140 mmHg, diastolic blood pressure ≥ 90 mmHg, or if the patient was using anti-hypertensive medication [10]. Diabetes was defined as a fasting blood glucose level of ≥ 126 mg/dL or a hemoglobin A1c of ≥ 6.5%, or if the patient was using treatment for diabetes [11]. Hypercholesterolemia was defined as a total cholesterol level of > 240 mg/dL or if the patient was using lipid-lowering agents [12], while obesity was defined as a body mass index (BMI) greater than 25 kg/m^2^ based on the World Health Organization’s recommendations for the Asian population [13].

### Measurements

Spirometry tests were conducted in subjects aged ≥ 40 years by using standardized equipment (model 1022; SensorMedics Corp, BD, Franklin Lakes, NJ, USA) according to the guidelines of the American Thoracic Society/European Respiratory Society [14]. Spirometry tests were repeated at least three times to ensure reproducibility and validity. The calculation for predicted values was based on the predictive equation for the Korean population [15]. To evaluate OA, radiography of the joint was performed in participants > 50 years of age using a SD3000 Synchro Stand (Accele Ray, Switzerland). Anteroposterior and lateral plain radiographs of the knee, hip, and spine were acquired. Radiographic changes related to OA in each joint were independently assessed by two trained radiologists using the Kellgren–Lawrence (KL) grading system [16, 17]. If, for the same case, the KL grades provided by the two radiologists differed, the higher grade was accepted. If the difference comprised more than one grade, a third radiologist was consulted and the grade concordant with the third grade was accepted. A radiographic knee KL grade ≥ 2 in one or both knees was defined as radiographic knee OA. In addition, all patients described their current symptoms related to the sites of pain. QOL was measured using the validated Korean version of the self-administered EuroQOL Five Dimensions Questionnaire (EQ-5D) [18].

### Statistical analysis

The KNHANES was designed using a complex, stratified, multistage probability-sampling model, and data were analyzed via the complex-sample design to represent the prevalence in the Korean national population using SAS version 9.3 and R version 3.6.0. Data were presented as mean ± standard error, or frequency (%). In order to compare the characteristics of each subgroup, generalized linear regression was used for continuous variables and logistic regression was used for categorical variables. Models testing for an association between OA and diseases, including asthma and COPD, were adjusted for age, sex, BMI, and current smoking status (model 1), and were further adjusted based on whether OA medication was prescribed (model 2). Models testing for an association between the pain site or severity of OA, based on the radiographic KL grade, and each relevant disease were adjusted for age, sex, BMI, and current smoking status. A *P*-value < 0.05 was used to indicate statistical significance.

## Results

### Characteristics of the participants

**Fig. 1:**
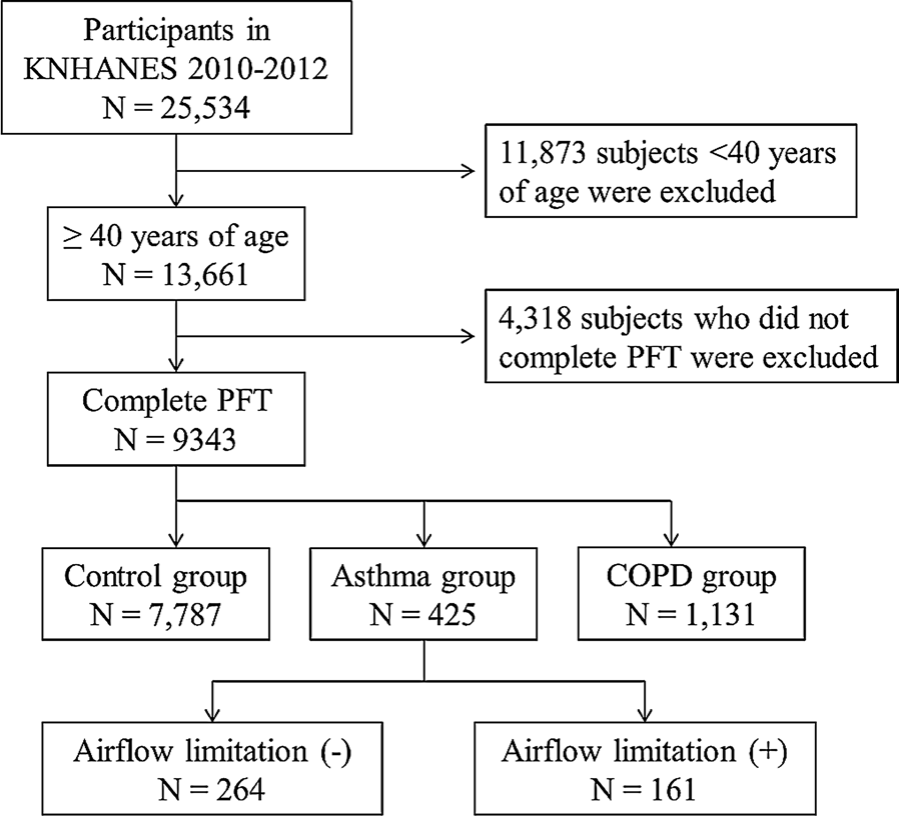
Flowchart describing recruitment of the study population. KNHANES, Korean National Health and Nutrition Examination Survey; PFT, pulmonary function test; COPD, chronic obstructive pulmonary disease

The completed questionnaire and spirometry data for a total of 9344 subjects aged ≥ 40 years from 2010 to 2012 were retrieved from the KNHANES. Among these, 425 patients had self-reported asthma (prevalence, 4.6% ± 0.3%), and 1131 had COPD based on the spirometric measurements (prevalence, 12.0% ± 0.5%). In the asthma group, 161 patients (prevalence, 1.7% ± 0.2%) showed airflow limitation on spirometry (Fig. 1 and Additional file 1: Figure S1). The demographic and clinical characteristics of patients with either one of the two respiratory diseases—asthma or COPD—were compared to those of the control group (Table 1). The age of the patients in the asthma group was higher than that in the control group, but lower than that in the COPD group. In comparison to patients with COPD, the asthma group included more females and more patients with obesity. Patients with asthma had comorbid hypertension more frequently than the control group, but less frequently than the COPD group. However, the prevalence of other metabolic syndromes, such as diabetes or hypercholesterolemia, did not significantly differ from that in the control group. Patients with asthma presented with the lowest FVC, and those with COPD had the lowest FEV_1_. In addition, QOL measured by the EQ-5D was the lowest in the asthma group (Table 1).

**Table 1:** Baseline characteristics comparing patients with asthma and COPD with control groups

	Controls (N = 7787)	Asthma (N = 425)	COPD (N = 1131)	*P-value*		
				Control vs. Asthma	Control vs. COPD	Asthma vs. COPD
Prevalence, %	83.4 ± 0.6	4.6 ± 0.3	12.0 ± 0.5			
Age, mean ± SD	54.2 ± 0.2	60.6 ± 0.9*	64.7 ± 0.4*	< 0.001	< 0.001	< 0.001
Male sex, %	44.0 ± 0.6	43.8 ± 2.9	76.3 ± 1.7*	0.93	< 0.001	< 0.001
BMI, kg/m^2^	24.4 ± 0.04	24.4 ± 0.2	23.6 ± 0.1*	0.98	< 0.001	0.001
Obesity, %	39.5 ± 0.7	41.4 ± 3.1	29.6 ± 1.8*	0.82	< 0.001	0.01
Smoking, %				0.27	< 0.001	< 0.001
Never	58.6 ± 0.7	53.7 ± 2.9	26.1 ± 1.7			
Ex-	20.6 ± 0.6	23.1 ± 2.7	40.3 ± 1.8*			
Current	20.8 ± 0.7	23.2 ± 2.7	33.6 ± 1.7*			
Comorbidities, %						
Hypertension	37.7 ± 0.8	49.4 ± 3.4*	53.2 ± 1.8*	0.05	< 0.001	0.24
Diabetes	11.2 ± 0.5	13.2 ± 1.9	17.1 ± 1.4*	0.50	< 0.001	0.06
Hypercholesterolemia	18.0 ± 0.6	20.1 ± 2.6	16.2 ± 1.5	0.41	0.26	0.17
Osteoarthritis	16.2 ± 0.6	31.9 ± 2.8*	17.8 ± 1.5	< 0.001	0.48	< 0.001
OA medication	5.6 ± 0.3	13.5 ± 2.2*	5.8 ± 0.8	< 0.001	0.69	< 0.001
Pulmonary function						
FVC, L	3.53 ± 0.01	3.22 ± 0.06*	3.66 ± 0.04*	< 0.001	< 0.002	< 0.001
FVC, %	93.4 ± 0.2	88.8 ± 0.8*	91.5 ± 0.5*	< 0.001	< 0.001	0.004
FEV_1_, L	2.81 ± 0.01	2.27 ± 0.05*	2.34 ± 0.03*	< 0.001	< 0.001	0.24
FEV_1_, %	94.6 ± 0.2	82.7 ± 1.1*	79.8 ± 0.5*	< 0.001	< 0.001	0.02
FEV_1_/FVC, %	79.8 ± 0.1	70.3 ± 0.7*	63.9 ± 0.2*	< 0.001	< 0.001	< 0.001
EuroQOL						
EQ-5D index	0.94 ± 0.002	0.88 ± 0.01*	0.92 ± 0.01*	< 0.001	< 0.001	0.003
EQ-5D VAS	75.6 ± 0.5	70.2 ± 2.5*	73.4 ± 1.4*	0.04	0.02	0.44

^*^indicates clinical significance (P < 0.05)

^**^P values were analyzed with control groups (Asthma & COPD group)

COPD, chronic obstructive pulmonary disease; BMI, body mass index; OA, osteoarthritis; FVC, forced vital capacity; FEV_1_, forced expiratory volume in 1 s, EQ-5D, EuroQOL Five Dimensions Questionnaire; VAS, visual analogue scale

### Association between asthma and osteoarthritis

**Fig. 2:**
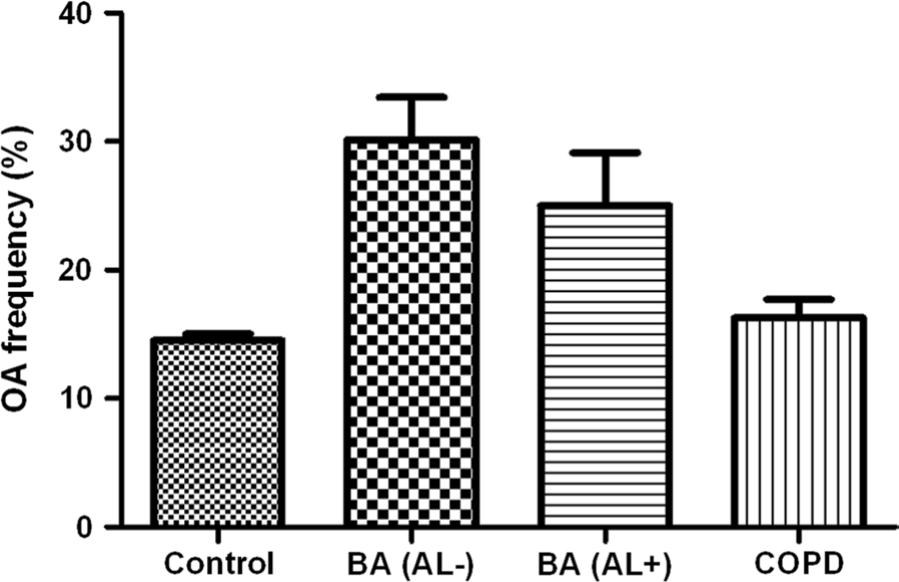
Prevalence of osteoarthritis. BA, bronchial asthma; AL, airflow limitation; COPD, chronic obstructive pulmonary disease; OA, osteoarthritis. Control vs. BA w/o AL, *P* < 0.001; Control vs. BA w AL, *P* = 0.021; Control vs. COPD, *P* = 0.48; BA w/o AL vs. BA w AL, *P* = 0.14; BA w/o AL vs. COPD, *P* < 0.001; BA w AL vs. COPD, *P* = 0.091

Among adults aged ≥ 40 years, 1190 patients (prevalence, 17.0% ± 0.5%) were diagnosed with OA by a doctor. The prevalence of OA was higher in patients with asthma (31.9% ± 2.8%) than in those with COPD (17.8% ± 1.5%) or the control group (16.2% ± 0.6%; Fig. 2). In the univariate analysis, OA was significantly associated with asthma (OR, 2.15; 95% CI, 1.70–2.72), but not with COPD (*P* = 0.34). The association between asthma and OA was significant regardless of the presence of airflow limitation on spirometry, although the effects were attenuated in patients with an airflow limitation (OR for asthma without airflow limitation, 2.36; 95% CI, 1.77–3.14; OR for asthma with airflow limitation, 1.72; 95% CI, 1.16–2.54). After adjusting for confounding factors such as age, sex, BMI, and smoking status (model 1), OA remained significantly associated with asthma (OR, 1.65; 95% CI, 1.27–2.13), but not with COPD (*P* = 0.40). To identify any effect of sex on this association, an interaction term was added to the previous multivariable model, but no significant interaction between sex and OA was noted (*P* = 0.43). Furthermore, to clarify the effect of obesity on this association, an interaction term between obesity and the presence of OA was added to model 1 and its interaction in the association with asthma without airflow limitation was not significant (*P* = 0.20). For sensitivity analysis, we reanalyzed the data, excluding patients with obesity, and the association between asthma and OA remained significant (OR, 1.70; 95% CI, 1.07-‍2.70).

To examine the influence of OA medication on asthma, the multivariable analysis also included the current medical treatment for OA with adjusted variables (model 2). The presence of OA remained significantly associated with asthma (OR, 1.56; 95% CI, 1.10–2.20), independent of the use of OA medication. Moreover, OA medication had no significant influence on the development of asthma (*P* = 0.63; Table 2). Patients with asthma were stratified according to the presence of airflow limitation in model 2—this association was only significant in patients with asthma without airflow limitation (Table 2). Since the development of airflow limitation could be affected by tobacco smoking, a sensitivity analysis that excluded current smokers was repeated and the presence of OA remained significantly associated with asthma (OR, 1.60; 95% CI, 1.11–2.31), independent of the use of OA medication (*P* = 0.64).

**Table 2:** Association of asthma and COPD with osteoarthritis

		Osteoarthritis	
	OR	95% CI	*P*
Asthma	1.56	1.10–2.20	0.013
Without Airflow limitation	1.97	1.32–2.93	< 0.001
With Airflow limitation	0.94	0.46–1.89	0.855
COPD	0.83	0.61–1.14	0.220

^*^Adjusted for age, sex, body mass index, and current smoking status

*COPD* chronic obstructive pulmonary disease

To determine the difference among the various sites of OA in relation to asthma, a subanalysis for each location of pain was performed. The prevalence of knee, back, and hip pain were higher in patients with asthma than that in the control group, but not in patients with COPD (Table 3). The results of the univariable analysis are summarized in Table 4. In the multivariable analysis, both knee and back pain were related to the presence of asthma, but hip pain was not significant (*P* = 0.30; Table 4). Among the 9344 individuals, joint radiography was performed for 6674, and the prevalence of radiographic knee OA using the KL grading system was 20.5% ± 2.9% in the asthma group, 9.2% ± 1.1% in the COPD group, and 12.1% ± 0.6% in the control group. Severe knee OA (KL grade 3 or 4) was also more prevalent in the asthma group than in the COPD or control groups (Table 3). The radiographic KL score for the knee joint was significantly associated with the presence of asthma in both the univariable and multivariable analyses (Table 4). The radiographic KL score for the lumbar spine was related to the presence of asthma in the univariable analysis, but lost significance in the multivariable analysis (*P* = 0.53). The radiographic KL score for the hip joint was not significant in either the univariable (*P* = 0.26) or multivariable analyses (*P* = 0.65). The associations between asthma and the radiographic KL scores for each joint are summarized in Table 4.

**Table 3:** Comparison of characteristics of affected joints

	Controls (N = 7787)	Asthma (N = 425)	COPD (N = 1131)
Knee pain, %	13.0 ± 0.5	26.0 ± 2.8*	14.6 ± 1.3
Hip pain, %	6.1 ± 0.3	10.5 ± 1.8*	6.5 ± 0.9
Back pain, %	14.8 ± 0.6	25.3 ± 2.6*	18.2 ± 1.5
Radiographic OAǂ	12.1 ± 0.6	20.5 ± 2.9*	9.2 ± 1.1*
Knee scale (KL grade)ǂ			
0	44.2 ± 1.1	36.2 ± 3.2	40.6 ± 2.0
1	22.9 ± 0.8	20.4 ± 2.8	27.3 ± 1.9*
2	13.0 ± 0.6	17.2 ± 2.7*	15.4 ± 1.4*
3	13.5 ± 0.7	17.2 ± 2.4*	11.4 ± 1.3
4	6.4 ± 0.5	9.0 ± 1.9*	5.3 ± 1.0

^*^Indicates clinical significance (P < 0.05)

ǂRadiographic evaluation was performed for the control (N = 5261), asthma (N = 353), and COPD (N = 1051) groups in subjects ≥ 50 years

COPD, chronic obstructive pulmonary disease; OA, osteoarthritis; KL grade, Kellgren–Lawrence grade

**Table 4:** Association of the location of pain or radiographic severities of osteoarthritis with asthma and COPD

	Asthma				COPD			
	Univariable		Multivariable		Univariable		Multivariable	
	OR	95% CI	OR	95% CI	OR	95% CI	OR	95% CI
Pain site								
Knee	2.25	1.80–2.80	1.78	1.40–2.26	0.92	0.77–1.10	0.78	0.64–0.96
Back	1.76	1.41–2.20	1.37	1.08–1.74	1.09	0.93–1.29	0.96	0.79–1.15
Hip	1.54	1.11–2.12	1.19	0.86–1.66	0.94	0.74–1.21	0.88	0.67–2.26
Radiographic KL grade								
Knee joint	1.19	1.10–1.29	1.10	1.01–1.21	0.97	0.92–1.03	0.94	0.88–1.00
Lumbar spine	1.25	1.08–1.46	1.05	0.89–1.24	1.49	1.36–1.64	1.15	0.99–1.25
Pelvis joint	1.16	0.90–1.49	1.06	0.82–1.38	1.51	1.30–1.75	0.95	0.81–1.12

*COPD* chronic obstructive pulmonary disease, *KL* Kellgren–Lawrence

## Discussion

The nationwide survey data collected from the general population revealed a significant correlation between asthma and OA. Despite the high prevalence of each of these conditions, there have been no reports describing their prevalence as comorbid conditions. Our study revealed the prevalence of OA in patients with asthma was as high as 31.9%. Moreover, the prevalence of OA was higher in patients with asthma compared to that in healthy controls or in patients with COPD, which is a comparative respiratory disease. The relationship between OA and asthma was remarkable in the group without airflow limitations, and the radiographic severity of the knee joint also correlated with asthma. The lower QOL in the asthma group may be a consequence of its comorbidity with OA in this population.

OA and asthma mutually affect each other through several mechanisms. First, asthma has an immunological and inflammatory pathogenesis which could simultaneously affect the development of OA. There is strong evidence that endogenous and exogenous reactive oxygen and nitrogen species play major roles in airway inflammation, which determines the severity of asthma [19]. The oxidative stress described above is also known to be an important factor in the development of OA [20, 21]. Genome-wide association studies (GWAS) have shown that several single-nucleotide polymorphisms of the gene encoding SMAD family member 3 (*SMAD3*) have been reported to be associated with knee or hip OA in both the Caucasian and Asian populations [22–24]. *SMAD3* is located on chromosome 15q21-22 and is known to have important anabolic effects on chondrocytes through intracellular messengers in the transforming growth factor-β signaling pathway [25, 26]. Remarkably, an epigenome-wide association study (EWAS) on asthma also reported differential methylation of inflammatory response–related genes, including *SMAD3* [27]. Second, medication for OA, such as NSAIDs, could influence the development of asthma, although the independent effect of such medication on asthma development was not significant in the present study. Our study suggests that the association between asthma and NSAIDs could be attributable to the effect of OA instead of NSAIDs themselves, but further larger studies are needed to confirm our findings. Third, asthma and OA have common features that are influenced by age, sex, obesity, and tobacco smoking—these might act as confounders, although we tried to adjust for these effects in multiple ways in the multivariable analysis.

This study revealed that the knee joint in patients with OA showed the most significant relationship with asthma. Since OA is a degenerative joint disease, its mechanism is primarily related to weight bearing and/or repetitive mechanical force; thus, the most commonly affected joints are known to be the hands and knees. However, the etiology of OA also includes the interaction between local tissue damage and the immune system, which may lead to chronic low-grade joint inflammation [28]. Altered levels of inflammatory mediators have been detected in OA synovial fluid suggesting synovial inflammation after meniscal damage [29]. Synovial joints are composed of diverse tissues that involve different loads, have distinct functional requirements, and possess differing proportional tissue types. The interaction of these factors may explain the preference of OA for certain anatomical sites, specifically the knees, hips, spine, hands, or feet [30]. Some reports have described strong genetic penetrance with diverse hereditary contribution, which is estimated to range from 30 to 65% depending on the joint sites [31–34]. Based on these studies, the association between asthma and OA, especially knee OA, could be explained, although we still cannot conclude whether there is a clear mechanism involved in this association.

The relationship between OA and asthma was found to be significant after adjusting for several confounding variables, and their association was more evident in the asthma group without airflow limitation. Asthma and COPD share similar phenotypic features and happen to be frequently misdiagnosed in clinical practice. Since patients with COPD may coexist in a group of patients with asthma with airflow limitation, the asthma group was split according to the presence of airflow limitation, and they presented distinct aspects of association with OA. In order to remove the patients with COPD that were part of the asthma group, we performed a sensitivity analysis excluding smokers and obtained a higher effect estimate.

Although the present study described the novel relationship between asthma and OA, the following limitations of the study should be considered to facilitate the interpretation of our results. First, the prevalence of asthma was calculated using results from a self-reported questionnaire, not by a provocation test, which created the risk of misclassifying patients. Moreover, owing to the absence of a provocation test, we could not compare the severity correlation of these diseases, such as the value of provocative concentration causing a 20% drop in FEV_1_. Although our study included patients diagnosed by doctors, misdiagnoses or underdiagnoses could have still occurred, especially in patients with asthma accompanying airflow limitation, with COPD. Therefore, we tried to stratify patients with asthma according to the presence of airflow limitation, and the association with OA was evident in a relatively pure asthma group without airflow limitation. Second, we had a less than optimum amount of information about the involvement of OA in each patient, including its severity, radiological and clinical information, and treatment information. Moreover, it is possible that the patients with severe OA and a poor health condition were not included in this study because they could not attend a nationwide survey. Additionally, among the participants aged ≥ 40 years, approximately 30% were excluded from the present analysis because they did not complete spirometry tests. Characteristic differences between these groups may affect selection bias. Finally, although OA medication alone was not associated with asthma in the subgroup analysis, we could not determine the exact type of medication used by the patients, such as steroids or NSAIDs. Further analyses are needed that focus on the differences based on the influence of the joint location and pattern of OA.

## Conclusions

The OA in patients with asthma has a high prevalence. OA and asthma can be considered as risk factors for each other. Considering their comorbid characteristics, these patients need special attention in terms of physical activity and QOL in clinical practice. Further larger studies, based on GWAS and EWAS, or intervention studies are needed to confirm our findings.

## Supplementary Information

**Additional file 1**. Composition of study population.

## Data Availability

The datasets generated and/or analyzed during the current study are publicly available through the Korean National Health and Nutrition Examination Survey webpage at https://knhanes.cdc.go.kr/knhanes/sub03/sub03_02_05.do.

## Abbreviations

BMI: Body mass index
COPD: Chronic obstructive pulmonary disease
EQ-5D: EuroQOL Five Dimensions Questionnaire
EWAS: Epigenome-wide association study
FEV_1_: Forced expiratory volume in 1 s
FVC: Forced vital capacity
GOLD: Global Initiative for Chronic Obstructive Lung Disease
GWAS: Genome-wide association study
KCDC: Korean Centers for Disease Control and Prevention
KL grade: Kellgren–Lawrence grade
KNHANES: Korean National Health and Nutrition Examination Survey
NSAIDs: Non-steroidal anti-inflammatory drugs
OA: Osteoarthritis
QOL: Quality of life
