# Supplementary figures and images for: Novel association between asthma and osteoarthritis: a nationwide health and nutrition examination survey
Source: BMC Pulm Med. 2021 Feb 16;21:59. doi: 10.1186/s12890-021-01425-6 (PMC7885236; doi:10.1186/s12890-021-01425-6)

**Supplements**

Supplemental Figure S1. Venn Diagram of study population


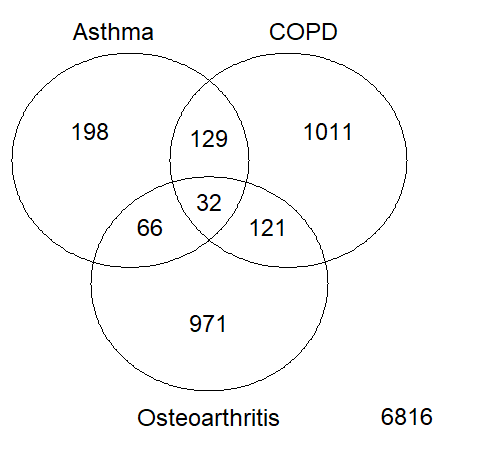

Supplement: Supplementary file 1 — Additional file 1. Composition of study population. [file 12890_2021_1425_MOESM1_ESM.docx]
